# Alemtuzumab Improves Cognitive Processing Speed in Active Multiple Sclerosis—A Longitudinal Observational Study

**DOI:** 10.3389/fneur.2017.00730

**Published:** 2018-01-16

**Authors:** Ester Riepl, Steffen Pfeuffer, Tobias Ruck, Hubertus Lohmann, Heinz Wiendl, Sven G. Meuth, Andreas Johnen

**Affiliations:** ^1^Department of Neurology, University Hospital Muenster, Muenster, Germany

**Keywords:** multiple sclerosis, immunomodulation, alemtuzumab, neuropsychology, cognition, neurocognitive disorders, magnetic resonance imaging

## Abstract

**Background:**

Several disease-modifying drugs have shown promising effects on cognitive impairment in multiple sclerosis (MS). Alemtuzumab, a humanized monoclonal antibody, is effective in controlling disease activity, however, has not been evaluated for its effects on cognition in detail so far.

**Objective:**

To explore the influence of alemtuzumab on cognitive impairment in active relapsing–remitting MS (RRMS) as well as possible clinical and neuroimaging predictors of cognitive changes during the first year of therapy.

**Methods:**

Extensive neuropsychological assessment was administered to 21 patients with active RRMS at baseline and again after the second treatment with alemtuzumab (mean time span: 15.05 months). Clinical and routine structural neuroimaging markers were explored for their capacity to predict individual courses of cognitive change.

**Results:**

Overall cognitive functioning remained stable or improved during the observational period of alemtuzumab treatment on average. Scores on two neuropsychological tests of processing speed significantly improved and clinically relevant individual gains of processing speed were seen in the majority of patients. Linear regression models showed that clinical and routine neuroimaging measures of disease activity could not fully account for these cognitive changes.

**Conclusion:**

Results suggest that alemtuzumab treatment in active RRMS stabilizes overall cognitive functioning and furthermore positively affects cognitive processing speed. Changes in processing speed were independent from clinical and structural neuroimaging parameters of disease activity and may thus represent an underrated and independent outcome measure to evaluate treatment effects.

## Introduction

Multiple sclerosis (MS) is a chronic inflammatory disease of the central nervous system, often affecting young adults (1). While cognitive impairment can be present in 40–70% of MS patients (2–4), it is not reflected sufficiently by the expanded disability status scale [EDSS (5)], the standard clinical measure for MS-related disability. Cognitive dysfunctions have a high impact on quality of life, may predict unemployment, and can negatively influence social activities (6). In relapsing–remitting MS (RRMS), deteriorations in information processing speed are regarded to be the primary cognitive deficit and are often accompanied by verbal memory and visuospatial deficits as well as executive dysfunctions (7, 8).

Disease-modifying drugs (DMDs) have been shown to slow disease progression and advances in physical disability (9). Nevertheless, cognition has been evaluated in detail in only a few DMDs so far. Regarding first-line treatments, interferon beta-1a has shown promising effects on processing speed and other cognitive domains (10–12). In line with their stronger effect on conventional clinical and paraclinical outcome measures, monoclonal antibody treatments (e.g., natalizumab and daclizumab-HYP) may even surpass first-line treatments regarding positive effects on cognition, particularly processing speed (13–16).

Alemtuzumab, a highly potent monoclonal antibody treatment, is approved in Europe since 2013 for patients with active RRMS. In randomized controlled trials, alemtuzumab improved relapse rate and disability and also slowed brain volume loss (17–19). In spite of these positive effects on disease activity, no study has so far explored the influence of alemtuzumab on cognition in detail. With the current longitudinal observational study, we sought to investigate the influence of alemtuzumab on a range of cognitive domains assessed with an extensive neuropsychological test battery. With regard to previously established positive effects of potent second-line treatments on cognition, we hypothesized that alemtuzumab may have a stabilizing or even positive effect on cognition, particularly for processing speed as the mainly impaired cognitive domain in MS (7).

Although previous studies showed the potential of DMDs to positively affect cognition, underlying factors that drive this cognitive change have not been clearly identified. We thus aimed to also explore predictors for cognitive changes under alemtuzumab treatment. For this purpose, clinical (EDSS, number of relapses, disease duration, and general intelligence at baseline) as well as routine structural neuroimaging markers [T_2_ lesion load, gadolinium-enhancing lesions (GELs)] at baseline were analyzed for their potential to predict individual cognitive outcomes.

## Materials and Methods

### Participants

Patients were recruited at the Department of Neurology at the University Hospital of Muenster between November 2014 and May 2016. All patients were diagnosed with MS according to the revised 2010 McDonald criteria (20) and were eligible for alemtuzumab treatment according to national guidelines and the recent alemtuzumab summary of product characteristics (SMPC). The treatment decision toward alemtuzumab was made independently of this study. The interval between prior treatment and alemtuzumab was according to national guidelines. None of the patients reported critical side-effects under his/her previous DMT and alemtuzumab treatment was initiated due to high or ongoing disease activity in all cases except one (here, treatment was switched from natalizumab because of increased risk for development of progressive multifocal leukoencephalopathy). As part of the clinical routine and according to national guidelines and the SMPC, all patients underwent clinical examination including determination of neurological status and disability as well as MRI of the brain and extensive laboratory examination. Eligible patients were then screened for participation in this study and gave informed consent upon enrollment. Patients with a history of psychiatric disorders, recent relapses (time span to last relapse >1 month) or neurological conditions other than MS were excluded. Clinical data including MRI imaging data were extracted from the medical charts of the patients. Ethical approval was given by local authorities (2014-398-f-S). Patients underwent baseline neuropsychological assessment at the screening visit prior to first alemtuzumab infusion. Follow-up assessment took place at the first visit after the second course of alemtuzumab [mean time span in months: 15.05 (SD = 2.06)].

### Procedure and Materials

The extensive neuropsychological test battery covered the domains of verbal learning, verbal and visual memory, attentional span, processing speed, visuoconstruction, and executive functions. Additionally, a measure of premorbid intelligence and questionnaires for depressive symptoms and fatigue were included. For details regarding the test protocol and cognitive domains, see Table 1.

**Table 1:** Neuropsychological tests and cognitive domains measured.

Cognitive domain	Neuropsychological test	Outcome measures
Verbal learning	Rey auditory verbal learning test (RAVLT)	RAVLT 1–5 total words learned

Verbal memory	Rey auditory verbal learning test (RAVLT)	RAVLT 6 recall after interference; RAVLT 7 delayed recall after 30 min.; RAVLT recognition 8

Attentional span	Digit span forwards	No. of digits memorized

Visual memory	Rey complex figure test (RCFT)	RCFT immediate recall

Visuoconstruction	Rey complex figure test (RCFT)	RCFT copy

Executive functions	Verbal fluency S-words and animals; Trail Making Test B (TMT-B); digit span backwards	S-words named during 1 min resp.; seconds needed for Trail B; no. of digits memorized

Processing speed	Trail Making Test A (TMT-A); Symbol Digit Modalities Test (SDMT); Rey complex figure test (RCFT)	Seconds needed for Trail A No. of correct responses in 90 s (written) Time to copy in seconds

Intelligence	Standard Progressive Matrices (SPM)	No. of correct responses

*German version of the (RAVLT) (21, 22), (RCFT) (23), Trail Making Test Part A and B (24) (TMT A and B), digit span forwards and backwards (25), verbal fluency S-words (26) (phonematic) and animals (26) (semantic), SDMT (27), SPM (28)*.

Test administration was conducted in accordance with the manuals in a quiet setting by an experienced neuropsychologist. Mean administration time of the test battery was 1.5 h. In order to avoid practice effects, alternative forms of neuropsychological tests were used for the follow-up testing whenever practice effects could be expected [Rey Auditory Verbal Learning Test (21, 22) (RAVLT), Rey Complex Figure Test (23) (RCFT)]. Test raw scores were standardized and transformed into normative percentile ranks controlling for age, sex, and education. Patients were classified as cognitively impaired when they scored beneath the fifth percentile rank in two or more cognitive measures.

### Statistical Analysis

Statistical analyses were conducted with SPSS Statistics 24 (IBM, New York, NY, USA); figures were created using Prism7 (GraphPad Software, CA, USA) and Microsoft Excel. Prior to employing statistical tests, test data were visually examined for skewness and outliers and checked for normality using the Kolmogorov–Smirnov test. Wilcoxon signed-rank tests were used to compare baseline and follow-up raw test scores and median percentile ranks. Alpha-levels were corrected for multiple comparisons using the Bonferroni-method (14 comparisons, corrected α = 0.0036). Median percentile ranks across all cognitive tests were computed for each patient at baseline and follow-up as a measure of general cognitive functioning. To evaluate individual courses of cognitive change, the number of patients with clinically significant impairment in two or more cognitive tests at follow-up was compared to baseline and tested for significance using McNemar’s tests. In order to explore predictors of this individual cognitive change (operationalized by subtracting the number of impaired tests at baseline from the number of impaired tests at follow-up), multiple regression models were employed. Commonly used clinical baseline parameters (EDSS, number of T_2_ lesions, number of GELs, relapse rate, disease duration as well as general intelligence) were added to this regression model as potential predictors. In a second model, the baseline-to-follow-up differences in EDSS, T_2_ lesion load, GELs, and relapse rate were also investigated as potential predictors of cognitive change.

## Results

### Demographic and Clinical Data

Demographic and clinical characteristics at baseline and follow-up are summarized in Table 2. Twenty-one patients (10 females, mean age 32.38 with a range of 18–50 years) were included in the study. At baseline, the mean EDSS was 2.12 (SD = 1.34). MRI showed that patients had a mean of 33.71 (SD = 23.28) T_2_ lesions and 4.67 (SD = 7.39) GELs [mean proportion of GEL/T_2_ lesions: 0.11 (SD = 0.14)]. Self-reported depressive symptoms on Beck’s Depression Inventory II (29) were mild with an average score of 9.62 [SD = 6.63; BDI-II; cut-off >18.5 for depression in MS patients (30)]. The mean fatigue score as assessed by the Wuerzburger Fatigue Inventory for MS (31) (WEIMuS; cut-off >32) was 27.72 (SD = 13.6) indicating mild self-reported fatigue in our sample.

**Table 2:** Means of demographic and clinical data at baseline and follow-up with *p*-values of Wilcoxon signed-rank tests.

Demographic data		Range			
Female, *n* (%)	10 (50.00)	N/A			
Education in years^a^ (SD)	11.48 (1.25)	9–13			
Age in years (SD)	32.38 (8.72)	18–50			
Follow-up period in months (SD)	15.05 (2.06)	12–19			
Disease duration in years (SD)	4.98 (5.44)	0–21.3			

**Clinical data**	**Baseline**	**Range**	**Follow-up**	**Range**	***p***

EDSS (SD)	2.12 (1.34)	0–4.5	1.88 (1.53)	0–5	0.410
No. of relapses (SD)	2.76 (1.97)	0–6	2.90 (2.14)	0–7	0.083
Mean relapse frequency^d^ (SD)	1.38 (0.99)	0–3	0.14 (0.36)	0–1	<0.001
No. prior treatments (SD)	1.81 (1.69)	0–7	N/A		N/A
Prior treatment		N/A	N/A		N/A
Naïve	6				
Beta-interferon	3				
Glatiramer acetate	2				
Dimethyl fumarate	3				
Fingolimod	4				
Natalizumab	3				
Months between treatments^e^ (SD)	3.67 (4.55)	0–18	N/A		N/A
No. T_2_ lesions	33.71 (23.28)	7–80	34.38 (24.06)	7–81	0.034
No. GELs	4.67 (7.39)	0–25	0.19 (0.40)	0–1	0.002
GEL/T_2_	0.11 (0.14)	0–0.4	0.01 (0.02)	0–0.1	0.003
No. patients with TPO antibodies^b^	5	N/A	8	N/A	0.250
BDI-II raw score (SD)^c^	9.61 (6.63)	0–20	9.52 (11.04)	0–46	0.099
WEIMuS raw score (SD)	27.72 (13.6)	3–44	23.29 (19.47)	0–56	0.052

*Clinical data of patients with RRMS and Alemtuzumab treatment (*N* = 21)*.

*N, sample size; EDSS, Expanded Disability Status Scale; RRMS, relapsing–remitting multiple sclerosis; GEL, gadolinium-enhancing lesions; TPO, thyroid peroxidase antibody; BDI-II, Beck’s Depression Inventory-II (cut-off: 18.5); WEIMuS, Würzburger Fatigue Inventory for Multiple Sclerosis (cut-off: 32); N/A, Not applicable*.

*^a^Education levels were grouped into three levels by years of general schooling, job trainings as well as academic training*.

*^b^Compared using McNemar’s test*.

*^c^Available from N = 13 at baseline, N = 21 at follow-up*.

*^d^Mean relapse frequency of patients per year*.

*^e^Interval between most recent prior treatment and alemtuzumab administration (months)*.

### Clinical and Cognitive Outcome

During the treatment phase, EDSS improved from 2.12 (SD = 1.34) to 1.88 (SD = 1.53; *p* = 0.410). Three patients each had one relapse within the study period (hypesthesia, dysesthesia, sensorimotor symptoms). Mean relapse frequency per person/year decreased significantly from 1.38 (SD = 0.99) to 0.14 (SD = 0.36) at follow-up (*p* < 0.001). T_2_ lesion load increased to 34.38 (SD = 24.06; *p* = 0.034). The number of GELs significantly decreased from 4.67 (SD = 7.39) to 0.19 (SD = 0.40; *p* = 0.002) and also the proportion of GEL/T_2_ lesions significantly decreased (*p* = 0.003). No significant differences from baseline to follow-up were seen for mean depressive symptoms (*p* = 0.099) or fatigue (*p* = 0.052).

#### Overall Cognitive Change

The median percentile rank of 35.24 (*SD* = 22.42) at baseline improved to 42.96 (SD = 19.78) at follow-up (*p* = 0.086), indicating a statistical trend toward overall cognitive improvement.

**Figure 1:**
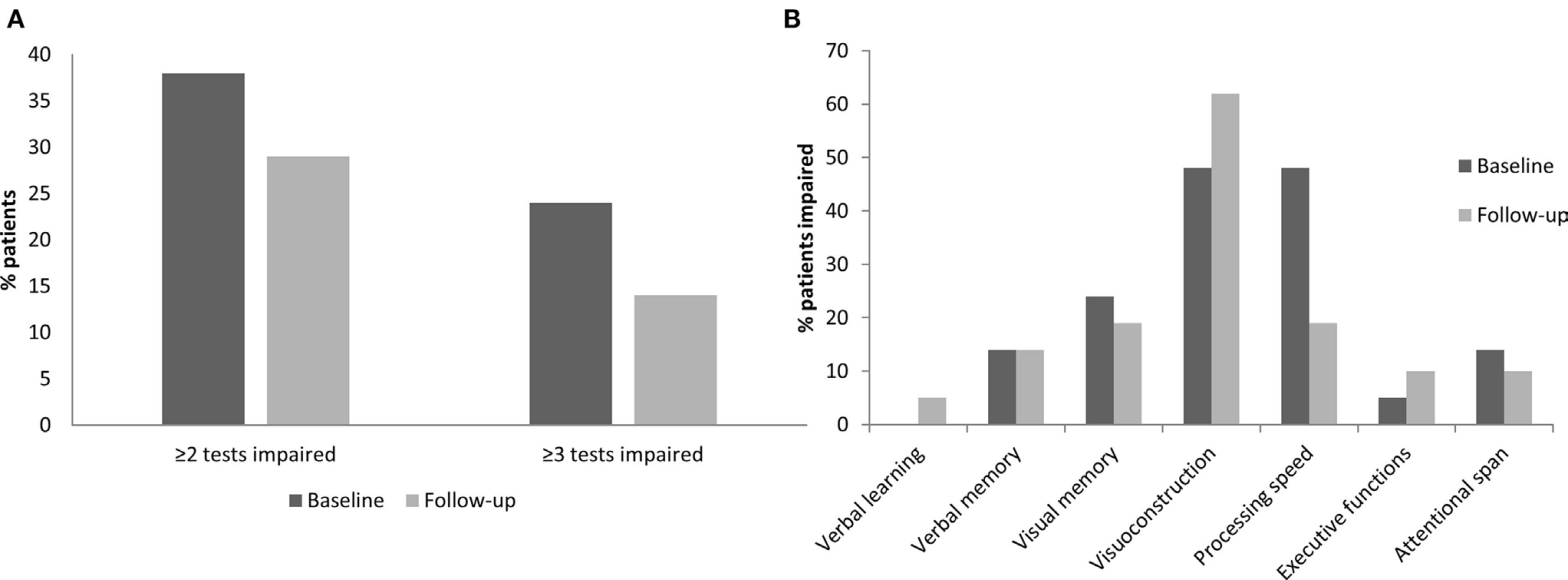
Cognitive change in overall cognition and in specific neuropsychological domains over the observational period of 15 months. **(A)** Percentage of patients with impairment in ≥2 tests and impairment in ≥3 tests at baseline and follow-up. **(B)** Percentage of patients impaired in each neuropsychological domain at baseline and follow-up.

In order to evaluate individual cognitive change, the number of patients with impaired test performance (cut-off point for impairment: <5th percentile rank) was also compared between baseline and follow-up (Figure 1A). At baseline, 8/21 patients (38%) were impaired in two or more neuropsychological tests and 5/21 patients (24%) were impaired in three or more tests. At follow-up, the number of patients impaired on two or more tests had decreased to 6/21 patients (29%) and the number of patients impaired in three or more tests decreased to 3/21 (14%). Using McNemar’s test for paired samples, these changes failed to reach statistical significance (all *p*s > 0.05).

#### Cognitive Change in Specific Domains

Figure 1B depicts the proportion of patients with cognitive impairment at baseline and follow-up in the different neuropsychological domains. At baseline, processing speed and visuoconstruction were the domains with the highest number of impaired patients; furthermore, patients showed impairments in visual and verbal memory, attentional span, and executive functions. At follow-up, a lower proportion of patients was impaired in the domain of processing speed [Symbol Digit Modalities Test (27) (SDMT), RCFT copy time, and Trail Making Test part A (24) (TMT-A)] when compared to baseline. This reduction was shown to be statistically significant by means of McNemar’s test (*p* = 0.031). No other domain showed significant changes between baseline and follow-up, and most importantly, no significant decrease in performance was seen under alemtuzumab.

#### Cognitive Change in Single Processing Speed Tests

**Figure 2:**
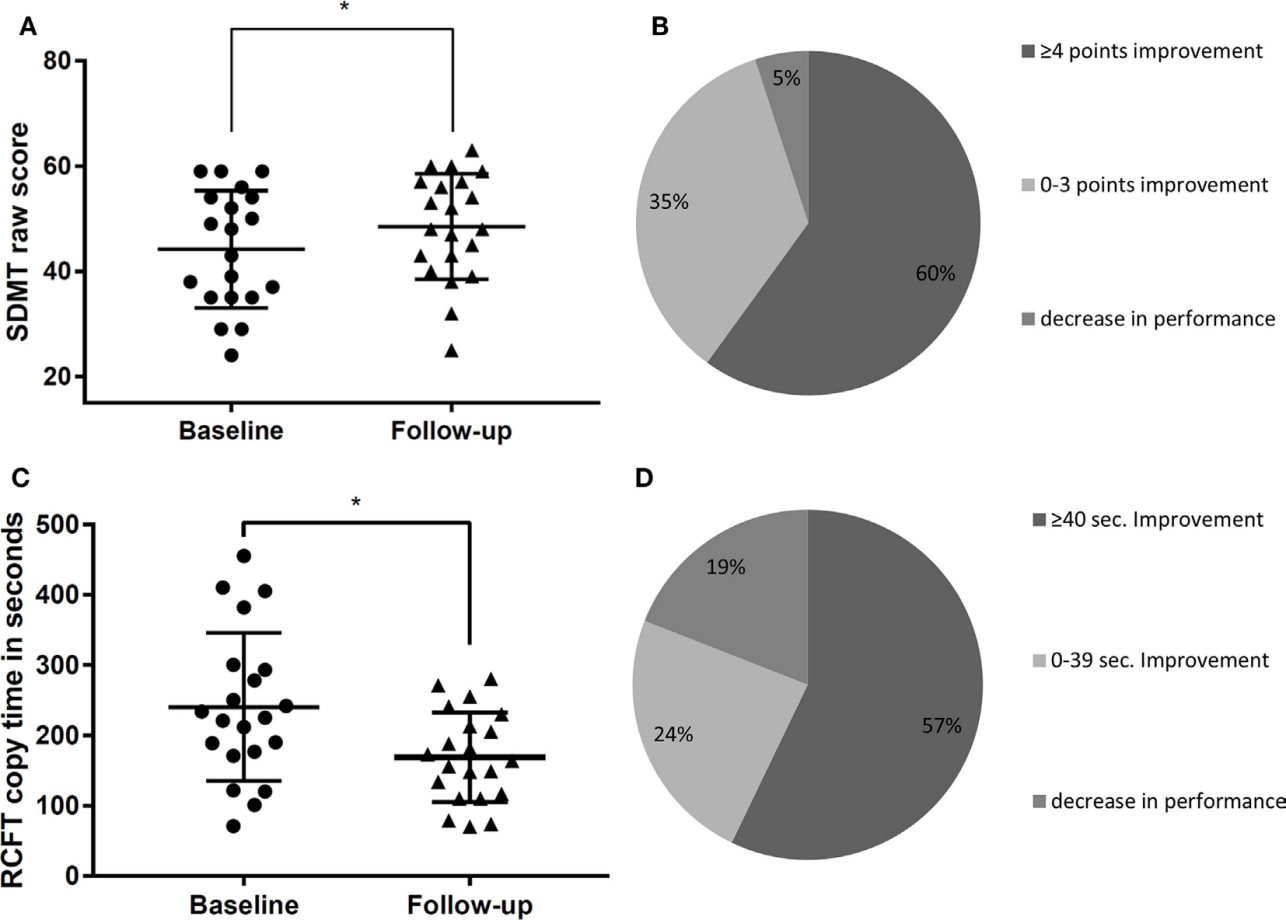
Cognitive change in processing speed tasks [Symbol Digit Modalities Test (SDMT), Rey Complex Figure Test (RCFT) copy time] at baseline and follow-up. **(A)** Individual scores of patients at baseline and follow-up on the SDMT, *Significance on alpha-level 0.0036. **(B)** Proportion of patients who showed a clinically significant change of ≥4 points, 0–3 points, or did not improve on the SDMT. **(C)** Individual scores of patients at baseline and follow-up on the RCFT copy time measure, *Significance on alpha-level 0.0036. **(D)** Proportion of patients who improved by ≥40 s, 0–39 s, or did not improve on the RCFT copy time measure.

Table 3 shows mean cognitive test results at baseline and follow-up testing grouped by neuropsychological domain. Consistent with our finding of a decrement of impaired patients in the processing speed domain, there was also a significant improvement in two single tests of processing speed. Mean patients’ scores on the written SDMT significantly improved from baseline to follow-up (*p* < 0.001; Figure 2A). This improvement can be regarded as clinically relevant in 12/21 patients, according to Morrow et al. (32), who defined an individually relevant improvement on the SDMT as four points according to the finding that a 4-point decrease on the SDMT was detectable in patients who advanced from employment to work disability within 3 years (Figure 2B). Similar to the SDMT, patients on average needed significantly less time on the RCFT time to copy condition during follow-up testing than during baseline testing (*p* = 0.002; Figure 2C). Improvement of the RCFT copy time was clinically relevant (as defined by an improvement of at least 40 s; Figure 2D) in 12/21 patients. Although no other test comparison between baseline and follow-up reached statistical significance, almost all mean test scores improved or remained stable over the course of observation (Table 3).

**Table 3:** Mean and SD of neuropsychological raw values at baseline and follow-up.

Cognitive domain	Cognitive measure	Mean baseline (SD)	Range baseline	Mean follow-up (SD)	Range follow-up	*p*-Value
Verbal learning	RAVLT 1–5	57.10 (9.22)	40–72	58.29 (11.29)	30–75	0.069

Verbal memory	RAVLT 6	11.70 (3.11)	5–15	11.33 (3.81)	1–15	0.917
	RAVLT 7	11.60 (3.60)	2–15	12.33 (3.00)	6–15	0.229
	RAVLT recog.	14.40 (1.05)	11–15	14.33 (1.49)	9–15	0.364

Attentional span	Digit span forwards	7.48 (2.56)	2–12	7.52 (2.04)	3–12	0.930

Processing speed	**RCFT time**	**240.38 (105.34)**	**71–455**	**168.95 (63.89)**	**70–280**	**0.002**
	**SDMT**	**44.20 (11.15)**	**24–59**	**48.52 (10.05)**	**25–63**	**<0.001**
	TMT-A	27.95 (9.88)	13–44	27.80 (9.25)	16–50	0.662

Visuoconstruction	RCFT copy	31.62 (4.23)	18–36	30.43 (3.18)	21.5–33	0.071

Visual memory	RCFT recall	20.12 (6.67)	8.5–31	21.69 (6.38)	7–34.5	0.590

Executive functions	Digit span BW	6.95 (2.33)	4–11	7.48 (3.49)	3–19	0.977
	Trail Making Test B (TMT-B)	66.85 (23.44)	34–120	64.69 (19.00)	29–107	0.641
	Phon. fluency	13.81 (5.23)	7–29	13.90 (5.22)	3–26	0.610
	Sem. fluency	22.95 (5.58)	10–37	24.14 (6.65)	11–40	0.256

*Wilcoxon signed-rank tests comparing the baseline cognitive function with cognitive function at follow-up in *N* = 21 RRMS patients with Alemtuzumab treatment. Cognitive tests in bold show a significant performance increase at level α = 0.036 (Bonferroni correction for number of comparisons)*.

*RAVLT, Rey auditory verbal learning test; digit span: digit span forward; RCFT, Rey Complex Figure Test; TMT-A, Trail Making Test part A; SDMT, Symbol Digit Modalities Test (written); Digit Span BW, Digit span backwards; TMT-B, Trail Making Test part B; Phon. Fluency, Phonematic verbal fluency with letter S (1 min.); Sem. fluency, Semantic verbal fluency with animals (1 min)*.

### Predictors of Cognitive Change

#### Baseline Predictors of Cognitive Change

In order to test whether cognitive change could be predicted by clinical routine parameters at baseline, EDSS, number of T_2_ lesions, number of GELs, number of relapses, disease duration, and general intelligence were analyzed for their influence on the course of cognition by means of a multiple regression model. The dependent variable was the difference between the number of impaired tests at baseline and follow-up. The proportion of explained variance in this model was *R*^2^ = 0.27. None of the included predictors reached statistical significance (all *p*s > 0.05).

#### Clinical Change as Predictor of Cognitive Change

In order to explore whether individual cognitive change from baseline to follow-up was fully dependent on changes in clinical parameters, baseline-to-follow-up difference scores of EDSS, T_2_ lesion load, number of GEL, and relapse rate were again explored as potential predictors using a multiple regression model. The proportion of explained variance of the model was *R*^2^ = 0.10. None of the entered variables were significant predictors of cognitive change (*p*s > 0.05).

## Discussion

### Outcome under Alemtuzumab

The present study provides a comprehensive and systematic analysis of the early changes in cognition following alemtuzumab treatment in active RRMS. Alemtuzumab had a positive effect on disease activity, and at least stabilizing effects on cognition within the first 15 months of therapy in patients with active MS. During the treatment period, the median percentile rank of all cognitive measures showed a trend toward improvement. Additionally, the proportion of patients impaired in ≥3 tests was reduced from 24% at baseline to 14% at follow-up, even though this result did not reach statistical significance, possibly due to small sample size and low statistical power. Reduction of cognitive impairment was mainly driven by overall gains regarding information processing speed, which improved significantly. Our findings provide further evidence that highly potent DMDs can stabilize and possibly even reduce cognitive impairment in active RRMS. Effect-sizes for cognitive outcome measures from this study cannot be directly compared to those of other DMDs due to broadly varying methodology. Several previous studies have shown that cognitive impairment increased slightly or remained stable (i.e., the percentage of cognitively impaired patients slightly increased from 21.4 to 21.6%) over the course of 2 years under interferon treatment (33–35). Thus, effects on overall cognition under alemtuzumab observed in the present study may be larger and rather comparable with those of natalizumab seen in previous studies, one of which reports a similar reduction in the percentage of cognitively impaired patients from 29 to 19% after 1 year (16, 36).

In line with previous results, patients showed pronounced impairment in specific cognitive domains already at baseline, i.e., in processing speed, verbal and visual memory, visuoconstruction, executive functions, and attentional span. Interestingly, alemtuzumab had a pronounced significant positive effect on information processing speed, the primarily impaired neuropsychological domain in MS (7). At follow-up after 15 months, patients had improved significantly in both the SDMT and the RCFT time to copy condition when compared to baseline underlining the validity of a general and overarching improvement in processing speed tasks during the observational period. Processing speed, as commonly measured by the SDMT or Paced Auditory Serial Addition Task (37) (PASAT-3), also primarily improved under treatment with other DMDs (12, 14–16, 38). It may be concluded that significant improvement in information processing speed is the most essential and driving factor of DMD-related cognitive stabilization or improvement.

While previous studies evaluating cognitive change under DMDs faced the critique of lacking clinical relevance, the changes seen in the SDMT in our sample were clinically relevant in 12/21 patients according to the conventions proposed by Morrow et al. (32). The SDMT is the most frequently employed neuropsychological test for processing speed in MS, has been shown to predict employment status, and correlates with activities of daily living (39, 40). Even though our study sample mainly consisted of young, well-educated adults in employment, it represents a group of patients with active RRMS carrying high risks of cognitive decline. Generally, patients with RRMS show declines in cognitive functioning over the course of the disease, especially during the first 5 years after symptom onset (41, 42). Alemtuzumab, as a potent monoclonal antibody treatment, is administered to patients who show high disease activity despite first-line treatments or initially exhibit a highly active disease course, consequently carrying high lesion load and brain atrophy (43, 44). Previous literature shows that high lesion load in the long run and brain atrophy leaves patients more vulnerable to cognitive decline (43, 45). For patients with active RRMS, improvement in information processing speed, e.g., as measured by the SDMT, may be relevant to quality of life and employment status (39, 46, 47).

### Predictors of Cognitive Change

Disease-modifying drugs may stabilize and even improve clinical as well as cognitive outcome measures in MS patients (9–16, 38, 48). Nevertheless, previous studies did not comprehensively analyze which factors at baseline can predict this stabilization and improvement. The present study aimed at exploring standard clinical baseline parameters as potential predictors of cognitive change; however, disease severity, duration of symptoms, number of relapses, T_2_ lesion load, GELs, and general intelligence at baseline could not predict overall cognitive changes under alemtuzumab treatment during the present short observational period of 15 months. Similarly, cognitive change was shown to be widely independent of clinical improvement during the study period by regression analyses (difference scores between baseline and follow-up of EDSS, relapse rate, T_2_ lesions, and number of GELs). Thus, the improvement of overall cognition cannot solely be explained by improvements on clinical parameters in our study sample.

Previous results regarding general associations between cognitive impairment and early disease severity markers in MS are contradictory. Disease severity and duration have been shown to only have a weak correlation with cognitive dysfunction in single studies (2). The EDSS may have issues with sensitivity of change and reliability (49). T_2_ lesion burden has previously shown to be less correlated with cognitive impairment than brain atrophy (50, 51). Volumetric analyses show promising associations with cognitive abilities in MS (52, 53). Particularly, global and deep gray matter atrophy or diffuse damage of gray and white matter seem more closely linked to progression of cognitive impairment in early MS (45, 54). Literature suggests a relationship between brain volume and measures of processing speed, i.e., SDMT and PASAT-3 score (52, 55, 56). For natalizumab, a protective role has been suggested for cortical gray matter volume during a course of 3 years that may be associated with a reduced rate of cognitive deterioration (13, 16). Similarly, the potent anti-inflammatory effect of alemtuzumab positively affects structural brain integrity parameters in patients with active RRMS. Alongside, outcome measures of reduced neuroinflammation, patients treated with alemtuzumab showed slowed brain volume loss (17, 19, 57). These effects on brain imaging parameters may be linked with improvement of cognition especially for processing speed, as this domain is closely associated with global brain atrophy (58). Automated structural volumetric imaging procedures, however, have not been established in clinical routine yet and were not evaluated in this study. The predictors tested in this study might not be suited for prognostic statements of positive or negative cognitive outcome under alemtuzumab. To conclude, cognitive change seems to be independent of the routine clinical parameters assessed under treatment with alemtuzumab and may thus be an underrepresented but potentially important outcome measure for treatment effects in future clinical trials.

### Limitations

Some methodological limitations have to be considered when interpreting our results. The present study is limited by a small sample size and may have been underpowered to detect standard clinical and neuroimaging markers that predict cognitive change under alemtuzumab treatment. A second limitation is the absence of randomized control groups. A control group composed of clinically and cognitively matched MS patients receiving an alternative disease-modifying drug could account for non-treatment-related changes in cognition. Because the present monocentric study observed patients with highly active MS only, longitudinal recruitment of a control group showing comparable clinical and cognitive baseline characteristics was not feasible. Due to the high disease activity and lesion load, patients in the present sample were, however, at high risk for cognitive decline, especially within the first years of disease (41–43). Hence, treatment success may be deduced from stabilization of cognition under alemtuzumab without comparison to a control group. Future multicentric RCTs are warranted to confirm our results. Furthermore, future studies with longer observation periods are of interest to explore whether outcomes are maintained or whether further effects can be captured.

A neurologically healthy control group could account for practice effects after a similar time span to follow-up testing. Practice effects on neuropsychological tests are an issue, which has to be considered when testing repeatedly. This concern was handled through the administration of alternative test forms. Moreover, the testing at follow-up was conducted at an interval of 15 months after baseline testing, rendering our results robust to many previously reported practice effects. Especially the SDMT has been proven to be a highly sensitive measure, which is not prone to practice effects when retesting after 1 year (59). Regarding the SDMT, it has to be considered that the written form used in the present study may be dependent on motor function. However, none of the included patients reported or showed pronounced motor slowing of the hands in clinical examination.

### Implications and Conclusion

In conclusion, the present study is the first to show positive or at least stabilizing early effects of alemtuzumab treatment on cognition. Alemtuzumab stabilized disease progression and improved overall cognitive functioning, specifically processing speed within the observational period of 15 months. Cognitive improvement seems to be partly independent of clinical change under alemtuzumab, further emphasizing the need to test for cognitive impairment in active MS as a potentially relevant and independent marker of disease activity. Research has shown that cognitive changes in MS patients may be present even when the concept of “no evidence of disease activity” is fulfilled on the basis of clinical and neuroimaging data (60).

In order to validate and extend the findings of positive effects of alemtuzumab on cognition in active RRMS, future studies need to examine larger sample sizes, implement randomized control groups, and include a long-term follow-up. Additionally, future research needs to consider more advanced clinical and neuroimaging baseline characteristics (e.g., global atrophy, deep gray matter atrophy, diffuse white matter damage, and functional network connectivity) to better predict cognitive change under DMDs.

## Ethics Statement

This study was carried out in accordance with the recommendations of the local ethics committee (Aerztekammer Westfalen-Lippe) with written informed consent from all subjects. All subjects gave written informed consent in accordance with the Declaration of Helsinki. The protocol was approved by the local ethics committee (Aerztekammer Westfalen-Lippe).

## Author Contributions

ER: drafting the manuscript, analysis and interpretation of data, statistical analyses, acquisition of data, and study coordination. SP and TR: revising manuscript for content and acquisition of data. HL: study concept and acquisition of data. HW: revising manuscript for medical writing. SM: study concept and revising the manuscript. AJ: study concept, revising the manuscript, study supervision, and supervision of statistical analyses.

## Conflict of Interest Statement

The authors declare that the research was conducted in the absence of any commercial or financial relationships that could be construed as a potential conflict of interest.
